# Insights on Morphology and Thermal Stability of Hollow Pt Nanospheres by In Situ Environmental TEM

**DOI:** 10.3390/molecules30040792

**Published:** 2025-02-08

**Authors:** Josephine Rezkallah, Xavier Sauvage, Bernhard Witulski, Simona Moldovan

**Affiliations:** 1 Univ Rouen Normandie, INSA Rouen Normandie, CNRS, Normandie Univ, GPM UMR 6634, 76000 Rouen, France; josephine.rezkallah@u-picardie.fr (J.R.); xavier.sauvage@univ-rouen.fr (X.S.); 2 Laboratoire de Réactivité et Chimie des Solides (LRCS), CNRS UMR 7314, Université de Picardie Jules Verne, 80000 Amiens, France; 3 Laboratoire de Chimie Moléculaire et Thio-Organique (LCMT), CNRS UMR 6507, ENSICAEN, Université de Caen, Normandie Univ, 6 Bd. Maréchal Juin, 14050 Caen, France; bernhard.witulski@ensicaen.fr

**Keywords:** Pt hollow nanospheres, in situ environmental TEM, galvanic replacement synthesis, electron tomography, catalysts

## Abstract

The fields of catalysis and energy storage nowadays quote the use of nanomaterials with well-defined size, morphology, chemical composition, and thermal stability in the high-temperature range and under harsh conditions of reactions. We present herein an approach based on in situ environmental scanning transmission electron microscopy (STEM), combined with analytical STEM and electron tomography (ET), for the evaluation of the thermal stability of hollow Pt nanospheres under vacuum and high-pressure hydrogen environments. Spherical Pt hollow nanospheres (HNSs) with an average diameter of 15 and 34 nm were synthesized by a galvanic replacement-based procedure using either steep or continuous addition of Pt salts during synthesis. The as-synthesized HNSs exhibit complex 3D structures with shells of a few nm constituted by small Pt nanoparticles and marked by the presence of open channels. The thermal stability of Pt-based HNSs under TEM vacuum and 1 bar of hydrogen flow is reported by considering microstructural changes, e.g., the build-up of a continuous shell and its evolution until HNSs collapse at elevated temperatures (>500 °C). Experimental findings are discussed considering fundamental phenomenological issues, i.e., NP faceting, NP diffusion, and subsequent NP sintering, with respect to the behavior of the systems investigated.

## 1. Introduction

Due to their exceptional catalytic properties, metallic nanoparticles (NPs) are widely employed in heterogenous catalysis [1,2]. Apart from their electronic [3], crystallographic [4], and chemical characteristics defined by the metal itself, the NP surface area is another key parameter crucial for the catalytic performance. Controlling the size, the geometry, and accessibility to the active phase is therefore a key strategy to tailor the materials’ catalytic activity [5,6]. The size reduction from a few nanometers down to atoms and/or clusters remains, for instance, a huge challenge in terms of both synthesis and application [7,8,9]. For the synthesis, difficulties relate to chemical purity, dispersion, and accessibility to the active phase [10,11,12]. In addition, most of the catalytic reactions take place at high temperatures and under well-defined environments, requiring nanostructures with high thermal and chemical stabilities. In this context, a deep knowledge of the as-synthesized catalysts characteristics and their behavior in the course of the reactions is a prerequisite for applications.

Noble metal NPs are among the most versatile catalysts extensively employed for industrial applications from energy conversion [13,14,15] to chemical production [16] and automotive exhaust purification [17]. The high resistance of noble metals to harsh conditions has allowed to draw a complete picture of Pt particles’ behavior in the course of model reactions such as CO oxidation [18,19]. Pt NPs are widely utilized in proton-exchange membrane fuel cells (PEMFCs), where the Pt active phase is supported by carbon-based materials, such as graphite or amorphous carbon. The stability of such a composite system upon reaction dictates the performance of the PEMFCs, and it relates to the thermal and chemical stability of both the Pt NPs, the support, as well as their interconnection [20,21,22]. In most of the cases, the active phase is constituted by solid Pt NPs with sizes inferior to 100 nm [5,23,24] which are more or less uniformly dispersed onto the supports [25,26,27]. Their thermal stability correlates with the catalyst deactivation in the high-temperature range, such that an increase in the particles’ size [28] and therefore a reduction in the surface-active area is expected to diminish the catalytic efficacy [29]. One of the strategies to increase the active surface is the use of nanoreactors in which both the inner and outer surfaces are accessible and the surfaces are marked by the presence of active sites [30,31]. The use of hollow NPs has been proposed in recent years as an alternative to solid NPs [32] due to their high microstructural and chemical stability upon reactions [33], and these types of morphologies have proved their efficacy as catalysts for electrochemical reactions [24]. Combining the high chemical stability and catalytic efficiency of Pt NPs with the increased surface area conferred by a hollow-type morphology can be foreseen as a suitable solution for the design and the development of highly effective catalysts. Apart from the active crystalline facets of the Pt NPs, one of the most sensitive parameters controlling the catalytic activity is the accessibility to the active phase. In the case of the nanoreactor types of the active phase, the active surface can be strongly improved by the presence of open nanochannels within the particle’s shells, which would allow the access of the reagents to the inner active surface of the catalyst during the reaction. Therefore, in this study, the emphasis is placed on the design, synthesis, and the deep characterization of hollow nanoreactors under reactive conditions.

The high spatial resolutions attained nowadays in a routine manner in transmission electron microscopy (TEM) recommend the use of this approach to determine the size, shape, morphology, and crystallography of NPs, whereas their chemical composition can be explored by adapted spectroscopy methods, i.e., energy-dispersive X-ray spectroscopy (EDS). When complex 3D morphologies are considered, electron tomography (ET) has emerged as a methodology to provide information on the nanomaterial’s intimate details that cannot be ascertained from 2D projections alone. Characteristics like the presence of macro-, meso-, and/or micro-pores [34], accessibility to the porous network and/or the active phase, and the exact location of the active phase on the supports employed in catalysis can be achieved [35,36]. Apart from the high panel of imaging and spectroscopy information available in TEM, the adaptation and development of in situ methodologies able to furnish deep insights on the material’s behavior under the impact of various stimuli has been explored in recent years. The application of thermal constraints, for instance, has long been limited to material testing under the microscope vacuum, which can be non-relevant for applications, particularly in catalysis. To overcome this barrier, substantial efforts have been dedicated to the development of environmental solutions in TEM by the set-up of dedicated TEMs and dedicated environmental cells [37,38,39]. Moreover, recent developments have allowed the set-up of operando approaches in TEM in an effort to rely on the reaction efficiency with the microstructural changes occurring during the reaction [40].

We report here for the first time on the high microstructural stability of Pt hollow nanospheres (HNSs) under vacuum and hydrogen environments as addressed by in situ TEM. The galvanic replacement method proposed by Liang et al. [41] was used for the synthesis of Pt-based hollow nanospheres under two different modes: (i) the addition of a freshly prepared aqueous Co NP solution to an aqueous solution of H_2_PtCl_6_ in one batch to obtain Pt nanoparticles thereafter called Pt_s_; and (ii) the reverse continuous addition of an aqueous H_2_PtCl_6_ solution via syringe pump to a solution of freshly prepared Co NPs to obtain Pt nanoparticles thereafter called Pt_c_. We here employ advanced analytical TEM approaches under 2D to explore the morphology, crystallography, and chemistry of the HNSs as synthesized by the approaches (i) and (ii). ET was employed to explore the as-synthesized specimens to highlight 3D features such as the hollow sphere character and the accessibility to the inner voids, etc. The 2D and 3D characteristics are comparatively addressed for the two sets of Pt HNSs in an effort to identify common points as well as main differences between them in terms of HNS size, shape, morphology, chemistry, etc. The in situ TEM experiments carried out under two different environments (vacuum and hydrogen) evidence the Pt HNS microstructural stability and behavior upon thermal treatment up to 650 °C.

## 2. Results

### 2.1. The Microstructure of the As-Synthesized Pt-Based Nanospheres

#### 2.1.1. Pt_s_ Hollow Nanospheres (Synthesized by Adding Co NPs to a Pt(VI) Salt Solution)

**Figure 1:**
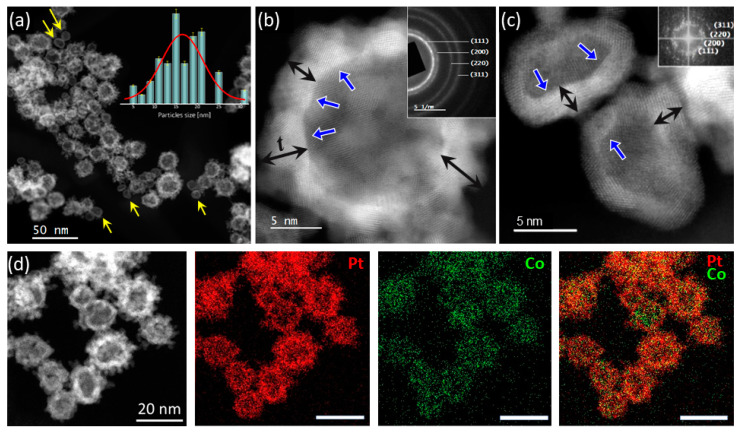
2D morphology and chemistry of the as-synthesized Pt_s_ HNSs. (**a**) STEM-HAADF micrograph, with the HNS diameter distribution histogram (yellow arrows: HNS with continuous shell), (**b**,**c**) HR STEM-HAADF image of individual HNSs (SADP and FFT of PtCo disordered phase) as insets, t denotes the shell thickness, whilst the blue arrows point to the inner HNSs facets (**d**) EDS elemental maps of HNSs and spatial distribution of Pt and Co.

The morphological and chemical characteristics of the Pt_s_ hollow nanospheres (HNSs) are displayed in Figure 1. The analyses reveal that most of the HNSs synthesized by this procedure are constituted by interconnected nanoparticles (NPs) with diameters of 1–2 nm assembled in a spherical hollow structure. The HNS size histogram (Figure 1a inset) shows the average outer diameter of the HNSs of 15 nm ± 2 nm (sizes from 5 to 30 nm). As compared with the data reported by Liang et al. [41], the mean outer diameter of the HNSs synthesized in the present work is about a half for similar morphologies. However, two types of nanoparticles have been identified in the current work: HNSs with irregular shell formed by small size nanoparticles (NPs) and HNSs with continuous shell (yellow arrows in Figure 1a). Small outer diameters up to 15 nm have been identified for the HNSs with a smooth and constant thickness of the shell (2–3 nm), whilst outer diameters higher than 10 nm are associated with nanospheres having interconnected NPs. For the latter, the mean shell thickness ranges between 2 and 5 nm (Figure 1), suggesting a random local superposition of up to three NPs in the radial direction. The inner HNS surface appears to be continuous, as revealed by the 2D projection images. Concerning the inner surface of the Pt_s_ nanospheres, the 2D projection images in Figure 1b,c reveal the presence of facets indicated by the blue arrows.

The high-resolution HAADF-STEM micrograph in Figure 1b clearly demonstrates the crystalline structure of small NPs forming the nanospheres, whereas the selected area diffraction pattern (SADP) recorded on a region with several tens of HNSs confirms the presence of the fcc Pt–Co disordered phase [42]. The chemical maps acquired on a representative region of this sample by energy-dispersive X-ray spectroscopy (EDS) displayed in Figure 1d shows that the Pt and Co distribution is uniform all over the volume of the HNSs. Furthermore, the quantitative analysis determines a mean Co content of 13 at. % ± 5 and a constant Pt/Co ratio within an isolated nanoparticle (see also Figure S1).

**Figure 2:**
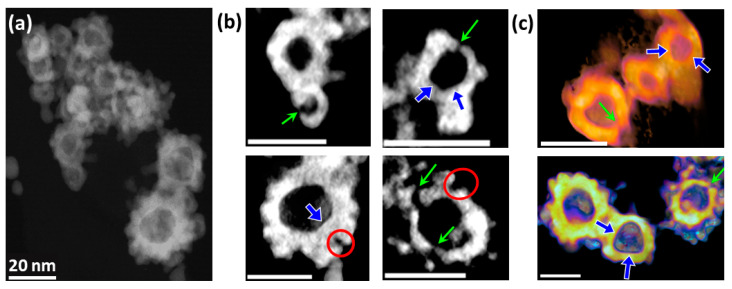
3D characteristics of Pt_s_ HNSs. (**a**) 2D projection acquired at −10° of the area chosen for the ET; (**b**) slices redrawn from the volume along random directions; (**c**) volume images extracted along random sections (green and blue arrows: HNS channels and inner surface facets; red circles for the surface topology); scalebar 20 nm.

Electron tomography (ET) carried out on a representative area within the Pt_s_ specimen confirms the hollow structure of the nanospheres and underlines their complex 3D morphology (Figure 2a). Accordingly, the HNSs with the apparent continuous shell shown in Figure 1b are constituted by interconnected NPs with diameters of about 2 nm packed in structures with an outer roughness up to the NPs’ diameters (red circles in Figure 2b). As anticipated by the 2D data, local faceting of the HNS inner surface occurs (blue arrows in Figure 2).

The most interesting feature revealed by the 3D tomographic investigation is the presence of open channels within the shell, for both morphologies. This hypothesis has been advanced by Liang et al. [41] for similar HNSs, but no proof has been provided in this direction. From a quantitative point of view, a mean number of 5 ± 2 channels have been identified for each HNS, whilst the opening diameter varies from 1 nm to 5 nm.

#### 2.1.2. The Pt_c_ Hollow Nanospheres (Synthesized by Continuous Addition of a Pt(VI) Salt Solution to Co NPs)

**Figure 3:**
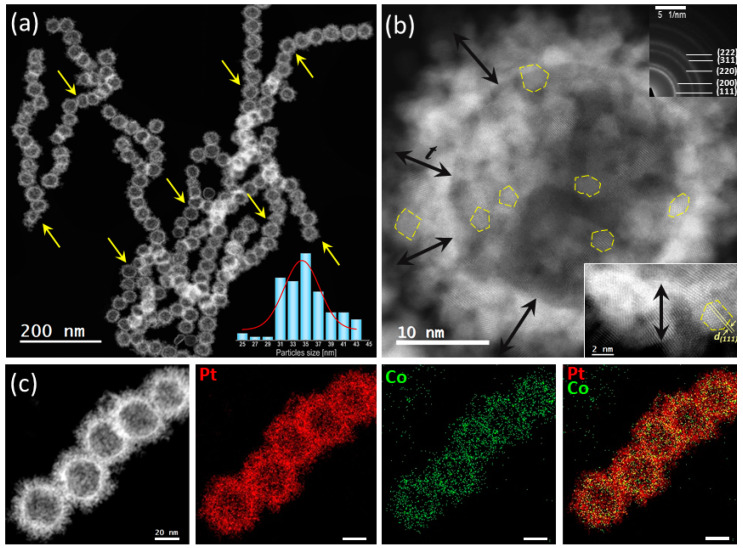
2D morphology and chemistry of the as-synthesized Pt_c_ HNSs. (**a**) STEM-HAADF arrangement of Pt_c_ HNSs in chains with the HNS diameter distribution histogram in inset (the yellow arrows point to the chains), (**b**) HR STEM-HAADFmicrograph of an HNS and the SADP as inset (crystallography PtCo disordered phase), t denotes the shell thickness and the dotted lines encircle NPs from the shell, (**c**) Pt and Co STEM-EDS elemental maps and their superposition.

The nanospheres synthesized by the second procedure—i.e., reverse continuous addition of an aqueous Pt(VI) salt solution to an aqueous solution of freshly prepared Co NPs—assemble in chains with lengths between 400 and 900 nm (Figure 3 and Figure S2). Statistically, the Pt_c_ HNSs investigated here are larger than the previous ones, with a mean diameter of 34 nm (from 25 to 44 nm), similar to the HNSs reported by Liang et al. [41]. The shell is constituted by interconnected small-size nanoparticles of about 2 nm with the same crystallographic features as for the Pt_s_ (disordered PtCo fcc phase) and well-defined nanofacets (yellow contours in Figure 3b), as confirmed by both the SADP and the HR STEM imaging (Figure 3b). The EDS analyses confirm the predominant presence of Pt and a small amount of Co uniformly distributed in the volume (7 ± 3 at. %).

**Figure 4:**
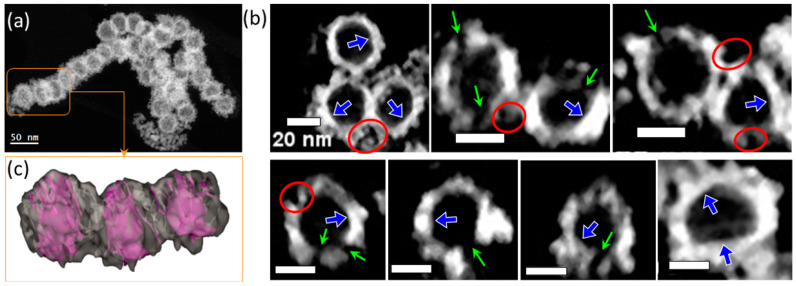
3D features of Pt_c_ HNSs (**a**); (**b**) slices redrawn from the volume along random directions (green arrows and blue arrows: channels and the inner surface facets; red circles: surface topology) (scalebar: 20 nm); (**c**) volume rendering after segmentation of the 3 HNSs encircled in (**a**).

Similarly to Pt_s_, the 3D analyses by ET (Figure 4) confirm the hollow morphology of the nanospheres and their complex nanostructuration. Here, the 2 nm interconnected NPs form a shell with a quasi-constant thickness from 4 to 8 nm. The HNS outer roughness is given by the size of the individual NPs or locally by the porosities resulting from the particles’ arrangements on the sphere surface (see the regions encircled in red). On the contrary, the HNS inner surface is smooth and locally exhibits facets (blue arrows). The open channels (green arrows in Figure 4b) are randomly distributed within the HNS shell and their widths range between 6 and 10 nm. The average number of channels per nanosphere is 14 ± 2.

### 2.2. Thermal Stability of Pt HNSs

Considering the multiple resemblances identified within the two sets of HNSs and for reasons of comprehensibility, only detailed data corresponding to the thermal stability of Pt_c_ HNSs will be extensively presented. A thorough comparative discussion on the behavior of the two sets of HNSs will be carried out in the Section 3, based on the data corresponding to the Pt_s_ HNSs provided in the Supplementary Information and used as a fundament for the comparative analysis.

The vacuum-assisted thermal treatment of the Pt-based nanospheres was carried out under the microscope vacuum (range of 10^−^^5^ Pa) and temperatures up to 550 °C. The hydrogen-assisted treatment was performed at 1 bar of H_2_ flow and temperatures up to 400 °C and 650 °C for the Pt_s_ and the Pt_c_ specimens, respectively. For reasons of comparison, microstructural changes were systematically followed by acquiring STEM-HAADF micrographs of the very same regions at well-defined temperatures and using the very same imaging conditions. The changes within such a region corresponding to the Pt_s_ HNSs under vacuum are shown in Figure S3 in the Supplementary Information. The analysis is based on the raw images from Figure S4, whereas the H_2_ treatment of the same specimen is compiled in Figure S6, by using the raw data in Figure S7 (see Supplementary Information).

The in situ TEM approach employed in this study enables the exploration of the microstructural evolution of Pt-based HNSs and the determination of the NPs’ sintering mechanisms under vacuum and hydrogen environments. In order to ascertain that the observed phenomena are not influenced by artifacts related to the electron beam, the areas monitored during all experiments have been compared to other regions not exposed to electron irradiation. As such, the HNSs from both regions were found to exhibit similar behavior, which is an indication that the electron beam has no notable influence on the phenomena observed in this study.

#### 2.2.1. Thermal Stability of Pt HNSs Under Vacuum

**Figure 5:**
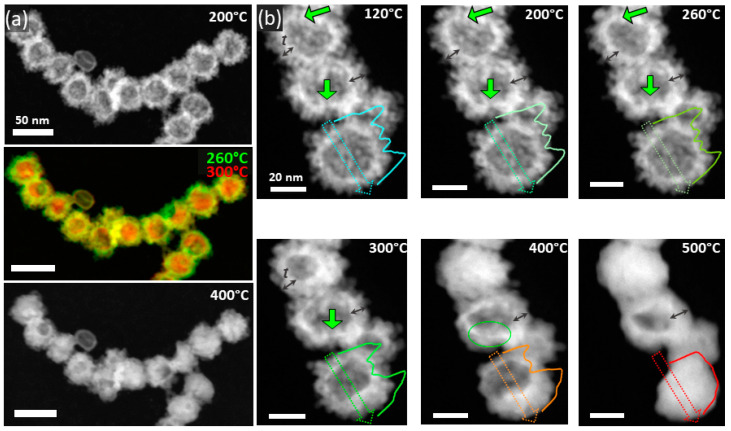
Vacuum-assisted high-temperature thermal treatment of the Pt_s_ HNSs. (**a**,**b**) STEM-HAADF micrographs of two regions acquired for temperatures from 120 °C to 550 °C (full series of (**a**) in Figure S5); intensity profiles as insets in (**b**) redrawn across one of the HNSs; the green arrows point to the HNS channels (scalebars: 50 nm and 20 nm).

Figure 5 depicts micrographs of two representative regions at different temperatures which were chosen to illustrate the phenomenological transformations occurring within the Pt_c_ HNS (see Figure S5 for detailed vacuum thermal treatment data). A thorough analysis of the micrographs acquired on several areas evidenced that up to 280 °C, the nanosphere morphology remains unchanged in terms of the morphological stability of the shell. The superposition of images acquired at 260 °C and 300 °C displayed in Figure 5a allowed the identification of the main changes. First, the “reorganization” of the NPs from the shell correlates with the increase in the mean size of the NPs, such that at 280 °C the NPs’ mean diameter reaches 4.3 nm, whereas at 300 °C the NPs’ size reaches about 5.7 nm and the shell broadens. The NPs’ coalescence continues up to 300 °C even more, such that a quasi-continuous shell forms at about 300 °C. A temperature increase up to 500 °C leads to the collapse of the individual hollow nanospheres. This manifests with the complete or partial (small number of HNSs) filling of the voids evolving already at 400 °C (Figure 5a). Notably, at 550 °C most of the HNSs become solid (see Figure S5). This phenomenon is accompanied by a diminution in the mean HNS sizes down to 30 nm at 550 °C (about 10% of the initial diameter), whilst at 300 °C the HNS mean diameter was reduced to 33 nm, as proved by the histograms displayed in Figure S10.

As one of the most important outcomes of this investigation is the presence of open channels within the shell, their morphology changes along the thermal treatment are important to study (marked by green arrows in Figure 5). The width of the channel evolves with the temperature from 1.5 nm at 120 °C to 3 nm at 300 °C. In terms of morphology, the channel evolution with increasing temperature is defined by the local neighboring environment such that we can assist at the diminution of the channel diameter or its blockage by the thermally activated motion of the NPs from the shell. As compared with the Pt_s_ HNSs, where a continuous shell forms at a lower temperature of 240 °C (Figure S6), in the case of the Pt_c_ HNSs the buildup of a completely continuous shell is less probable due to the higher number of open channels within the shell (Table 1).

In order to obtain deeper insights on the morphological changes, the intensity profiles extracted from the micrographs for the very same particle upon thermal treatment along the directions marked by the arrows are shown as insets. At 120 °C and up to 300 °C, the imprints of the shell and the projection of isolated NPs in the central part of the sphere are distinctively identified. At 300 °C and even more obviously at 400 °C, the peaks corresponding to the shell broaden and the difference between the intensities measured for the shell and the inner region diminish. Afterwards, at 450 °C the void is partially replaced by the metallic matrix forming a solid particle at 500 °C and beyond. As expected, the sizes of the HNSs evolve with the temperature, such that the HNSs’ outer diameter diminishes while increasing the temperature (Figure S10), as the thickness of the shell increases gradually until collapse. This behavior is proved by the broadening of the intensity profiles corresponding to both the shell and the inner region across two NPs from Figure 5b.

#### 2.2.2. Thermal Stability of Pt HNSs Under Hydrogen Flow

**Figure 6:**
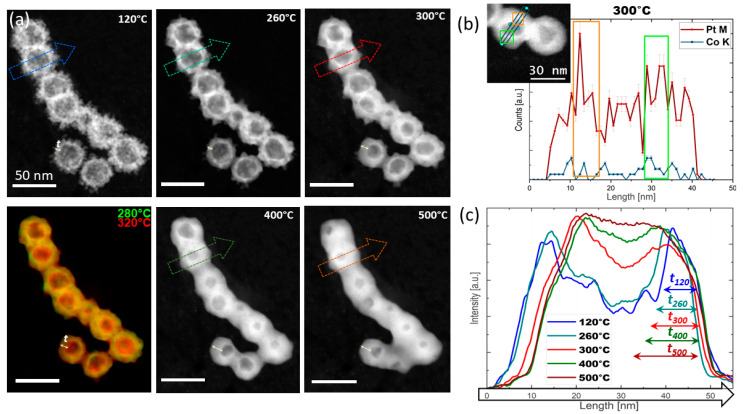
Hydrogen-assisted high-temperature thermal treatment of the Pt_s_ HNSs. (**a**) STEM-HAADF micrographs (full series in Figure S7) (t denotes the shell thickness (scalebar: 50 nm)); (**b**) EDS line profile analysis recorded at 300 °C along the direction in the inset; (**c**) intensity profiles along the arrows in (**a**) with the shell thickness (t) variation in inset.

Figure 6 shows the microstructural changes of a representative area within the Pt_c_ specimen from 120 °C to 500 °C (the full series is displayed in Figure S8). NPs located on the shell of the Pt HNSs slightly migrate and subsequently assemble to form a continuous shell at 260 °C for the large majority of the HNSs. A gradual decrease in the size of the central void within the HNSs is exhibited at 300 °C, with the diameter diminishing from 25 nm to approximately 18 nm at 300 °C. This continues with the increasing temperature, such that at 650 °C, cavities with sizes of 1–2 nm are identified for 80% of the HNSs (see Figure S8 for details). Similarly to the vacuum-assisted treatment, the inner surface of the HNSs becomes facetted (blue contours in Figure 6), but at a lower temperature of 240 °C. The high faceting rate and behavior is different as well as compared to the Pt_c_ HNSs (Figures S6 and S7) under similar treatment conditions. This is most probably a cumulated effect of the larger size and higher number of open channels for the Pt_c_ HNSs as compared to the Pt_s_ specimens.

A detailed analysis of the intensity profiles shows the presence of NPs within the shell, which slightly evolves with increasing temperature due to the NPs’ migration and subsequent coarsening. At 240 °C, the sphere core becomes smooth as the shell becomes continuous. The HNS inner and outer diameters diminish progressively with increasing the temperature as shown by the profiles in Figure 6c redrawn along the arrows in Figure 6a and highlighted by the corresponding shell thickness values (t) extracted at each temperature. Accordingly, an initial hollow sphere with an outer diameter of 45 nm finally becomes a solid particle with a diameter of about 35 nm, whereas the shell thickness increases from 5 nm to 15 nm. From a statistical perspective, the particle’s diameter reduces from an initial mean value of 34 nm to 31 nm at 300 °C and finally 28 nm at 550 °C, where most of the particles became solid (Figure S10). At the same time, apart from the morphology changes, the Z-contrast associated with the STEM-HAADF imaging indicates the Pt segregation on the HNS inner surface starting from 300 °C. Indeed, the STEM-EDS analysis confirms this segregation as two Pt peaks are identified on the inner surface rims along the profile line displayed in Figure 6b.

## 3. Discussion

### 3.1. The As-Synthesized Pt_S_ and Pt_C_ Hollow Nanospheres

Before delving into the mechanisms of the phenomena involved in the changes of Pt-based HNSs upon the in situ thermal treatment, it is worth highlighting the impact of the synthesis procedures on the HNS microstructure. The analysis of nanomaterials synthesized by the procedures adapted from Liang [41] reveals a series of similarities and differences between the two sets of HNSs prepared during this work, as compiled in Table 1.

The Pt-based particles exhibit hollow microstructures characterized by shells constituted by interconnected NPs with similar average diameter. The shell thicknesses are similar as well, with mean values slightly superior for the Pt_c_. The diameters of the NPs forming the shell as well as their connectivity define the external surface roughness, such that the HNSs of the Pt_c_ exhibit higher surface roughness than the Pt_s_. For the HNSs synthesized by the batch addition of Co NPs to the Pt(VI) salt solution (Pt_s_), the presence of two types of morphologies was identified: HNSs with a continuous smooth shell and HNSs with a rough outer surface. During this galvanic Pt HNS synthesis, no matter the order and speed of addition and/or the speed of stirring, the genesis of Pt nucleation centers on the outer surface of the Co NPs occurs. Under stirring, the growth of Pt NPs leads to nucleation of NPs with diameters up to 3 ± 1 nm with well-defined facets. At the same time, other nuclei form on the surface of the supporting Co particle, and they will serve as nucleation seeds for other NPs. From a statistical point of view, there is a high probability for the NPs to grow closely to each other, due to the presence of numerous nucleation centers randomly positioned on the surface of the Co particle. Obviously, a large number of nuclei will remain in their original state (size and shape) and in the close vicinity of the Pt NPs grown up to their final size. This leads to the genesis of a continuous Pt-rich layer constituted by both NPs and Pt-rich nuclei at the location of the Co particle’s surface. Under these circumstances, the presence of inner nanosphere facets right after the synthesis is not surprising as the facets appear to follow the morphology of Co supporting particles. Moreover, a faceted metal catalyst has more atoms located at edges and corners, where atomic density and coordination are much lower, profoundly enhancing the surface adsorption.

Both sets of particles contain residual Co, most probably from the templating particles. The residual Co identified by the chemical analysis shows no preferential location within the HNS, suggesting that the Co from the templating particles is progressively removed from the new created matrix. The Co partial removal leads most probably to the random or chain-like arrangement of HNSs for the Pt_s_ and Pt_c_ specimens, respectively. However, the amount of the residual Co is significantly higher in the case of the simultaneous addition of the source components (Pt_s_) than for the continuous incorporation of the initial constituents. The higher Co removal rate in the case of the Pt_c_ system can be at the same time correlated with the presence of a higher mean number of channels within the shell of the HNS, e.g., 14 as against five in the case of the Pt_s_, which serve as exhaust pipes for the cobalt. The presence of Co contaminations in both sets does not necessarily have a negative impact on their catalytic activity. Krajczewski et al. [43] have investigated the influence of these contaminations on the catalytic activity of the Pt nanostructures upon reduction of 4-nitrophenol by sodium borohydride in aqueous environments. These investigations confirmed that the enhanced catalytic activity of hollow Pt NPs is not solely due to their increased surface area, but as well to the catalyst’s geometric and electronic modifications associated to the presence of Co as a sacrificial template from the synthesis.

The most relevant difference between the two sets of HNSs relates to the average size of the nanospheres, in a way that the mean diameter of the Pt_c_ HNSs is about twice the one of the Pt_s_. This is most probably due to a slower and more controlled formation and sintering of Pt NPs during the slow but continuous addition of a Pt(VI) salt solution via syringe pump. The increase in diameter alone obviously acts as a thermal stability-increasing factor and is expected to lead to a reduction in the active surface area.

The presence of discontinuities within the particle shells, i.e., open channels, is by far the most striking and interesting output of this type of synthesis, particularly for the catalytic applications. The presence of channels within the shell has already been suggested by Liang et al. [41] but no valuable proof has been advanced so far. The origins of these features can be speculatively correlated to the galvanic process itself, if the growth of Pt nanoclusters/islands on the surface of the templating Co is associated to its slow dissolution by oxidation to Co2+. The growth of the Pt shell occurs at the expense of the Co core and therefore the presence of channels can be seen as a consequence of the synthesis procedure. During a reaction, the inner void of an HNS can be accessed by reactants through diffusion across the outer shell. From a quantitative point of view, in addition to the higher diameter, the Pt_c_ HNSs present a significantly higher number of channels (almost three times higher) than for the Pt_s_. The same trend is identified for the case of the channels’ mean diameters, with a mean channel opening of 8 nm as against 3 nm, corresponding to the Pt_c_ and Pt_s_ HNSs, respectively. The higher number of large channels is obviously attributed to the slow and controlled addition of the Pt(VI) salt to the Co NPs during the second synthesis, enabling the Pt(IV) to be more discretely and effectively reduced upon the sacrifice of the Co NPs into solution.

The accessibility to the active sites from the inner surface of the HNS systems, the topography of the outer surface with the presence of active sites, and the thickness of the shielding shell are the most important parameters to impact the rate and efficiency of a chemical reaction. The shell discontinuities and porosities are prone to facilitate the diffusion of reagent molecules into and outside the HNS volume [44]. At the same time, the thickness of the shell can have a certain impact on the duration of the reactant’s diffusion towards the active sites located in the inner surface of the HNS or of the reaction products to be released.

### 3.2. Mechanisms Controlling the Thermal Stablity of Pt HNSs

From a fundamental point of view, the NPs sintering can be associated to two distinct diffusion mechanisms: the Ostwald ripening (OR) characterized by the migration of individual atoms between stationary nanoparticles, and the particle migration and coalescence (PMC) involving the migration and fusion of entire nanoparticles. For the case of Pt HNSs, thermal treatment induces a “reorganization” of the NPs from the shell, and this correlates with an increase in the mean size of the NPs. This phenomenon is related to the Ostwald ripening process. As a consequence, the size of the NPs is expected to increase, triggering the increase in the shell thickness. From a practical perspective, one of the most employed methods to elucidate the mechanisms behind catalyst sintering is the particle-size distribution (PSD) [45,46,47,48]. However, it has been demonstrated that the coarsening mechanism of catalysts is influenced by numerous factors: the morphology and the shape of the NPs [49], the strength of interaction between the NPs, the support, and finally by the reaction environment.

From an experimental perspective, the current investigation is based on the STEM-HAADF micrographs captured at a gradual increase in temperature. Due to the complex geometry of this system, identifying the motion of individual NPs from the shell is far from obvious, and therefore only the enlargement of interconnected nanoparticles was targeted in a first approach. As such, the disappearance of certain NPs without mobility suggests that the sintering process is primarily driven by the Ostwald ripening mechanism. Several cases of NPs moving on the surface of the shell were however also identified.

If the hypothesis of coalescence by NPs migration and the coalescence mechanism [50] is advanced, we can use a simple approach to quantify the activation energy for NP coalescence. This is based on the measurement of the critical distance between two neighboring NPs that lead to coalescence (Figure S9). If an HNS is considered as a continuous support on which the NPs diffuse, we can estimate from the STEM micrographs the distances traveled by neighboring particles prior to their coalescence. The Arrhenius-type plot of this migration allows to estimate the activation energy. It is important mentioning here that the exact trajectory of these NPs’ motion is unknown due to the 2D image projection, and the measured distance can therefore be underestimated. By following this procedure, the activation energies for the diffusion of NPs of Pt_s_ HNSs were estimated at 307 meV and 172 meV for the vacuum and hydrogen-assisted thermal treatments, respectively. This large difference shows that NPs diffusion within the nanospheres’ shell occurs at lower temperatures under a hydrogen environment than in a vacuum. This is attributed to the relatively high reactivity of Pt to the reaction environment [51,52]. We are dealing here with the case of the NP self-diffusion on a particular geometrical configuration and for reasons of simplicity, the role of the geometry was neglected as the distance crossed by the NP is much lower than the NP diameter. Using Monte Carlo variational transition state theory and the Lennard–Jones interaction, Agrawal et al. [53] have estimated that the activation energies for the atomic Pt–Pt diffusion stands from 200 meV to 1.21 eV on the (111) and the (110) systems, respectively. As compared with the atoms’ diffusion on a well-defined surface, a higher value is expected for the E_a_ as in the current study we are dealing with 2 nm Pt NPs instead of Pt atoms. Indeed, in our case we have calculated that the energy of activation is superior to the atomic Pt diffusion but comparable with the activation energy of 2 nm Pt NPs’ diffusion onto graphene supports (297 meV), as determined by Moldovan et al. [54]. In the presence of H_2_, the E_a_ diminishes as expected, due to the high reactivity of Pt to the reaction environment.

The NPs’ diffusion leads to the reshaping of the HNS shells, such that the formation of a continuous smooth shell from the first stage is followed by the structural transformation of the hollow configuration into a solid one, phenomena associated with the collapse of the HNS. This restructuring is however kinetically constrained by the vacancies in the central void, which are correlated to the size of the inner void, as shown by Dubau et al. [55] who investigated the collapsing of Pt–Ni hollow nanoparticles under various atmospheres. This implies that the thermodynamics controls the structural reconfiguration of HNSs via the reduction in the total surface energy. Jiang et al. [56] used the MD simulations to classify the hollow nanoparticles as a function of their thermal stability in three classes: “stable” (no collapse), “half-stable” (partial collapse), and “unstable” (complete collapse) configurations. As applied to our systems, we can identify the three configurations as a function of the temperature of reference and of the environment used for the treatment. Moreover, we can clearly identify the critical temperatures at which the most important microstructural changes occur.

**Figure 7:**
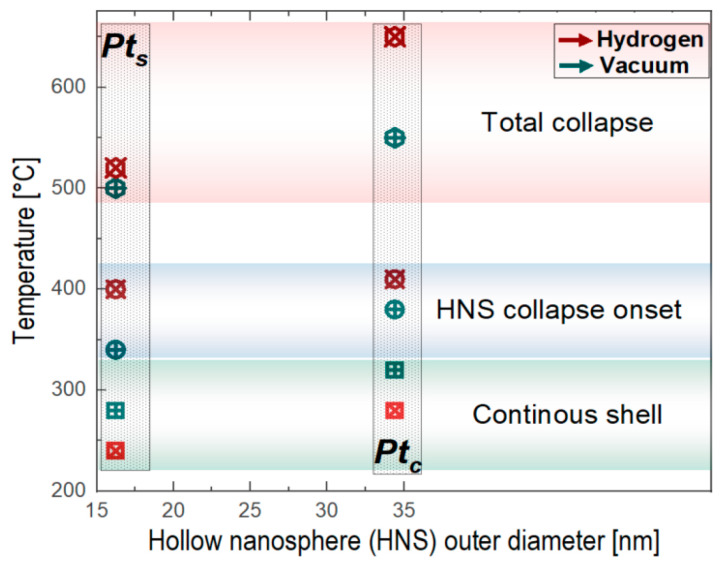
Synthesis diagram of the critical temperatures identified by the in situ treatment of the HNSs under vacuum and hydrogen environments.

The two sets of HNSs can be compared by considering the temperatures at which the continuous shell is “complete”, the HNS collapse initiates, and the solid NP forms (Figure 7). The critical temperatures corresponding to the Pt_s_ are generally lower than those for Pt_c_ HNSs, which is consistent with the morphological observations showing that the mean outer diameter of the Pt_s_ HNSs is about a half (16 nm) of the Pt_c_ HNSs (34 nm). For both types of HNSs, the continuous shell forms at lower temperatures upon hydrogen treatment, which is mainly due to the high reactivity of Pt to the H_2_ environment.

For the complete collapse, the balance completely changes, in a way that the vacuum-assisted treatments occurs earlier than under H_2_, no matter the HNS diameters. This might be surprising at a first glance, but in this case we are dealing with complex 3D systems in which the inner surfaces are accessible to the reactive gas, which leads to a higher degree of inner surface facetting under H_2_ than for the vacuum treatment. On the one hand, it is well known that the presence of facets leads to the increase in nanoparticles’ chemical and microstructural stability [57]. In addition, the Pt reactivity to H_2_ and the high temperature act as favorable conditions for the Pt facets to form. The presence of channels within the shell, on the other hand, ensures the access of the hydrogen to the HNS inner surface. As an effect, the inner surface facetting occurs and evolves as the temperature increases, leading to the genesis of a rather stable hollow HNS with facetted inner surfaces.

## 4. Materials and Methods

### 4.1. Synthesis of the Hollow Pt Nanospheres Pt_s_ and Pt_c_

The synthesis of hollow Pt nanospheres followed the galvanic procedure proposed by Liang [9]. It was modified with the aim to achieve different sizes, morphologies, and compositions of the hollow Pt nanospheres Pt_s_ and Pt_c_.

Synthesis of Pt_s_: The reaction was performed in a 100 mL 3-necked flask, sealed with rubber septa and equipped with a magnetic stirring bar. The flask was placed above a magnetic stirring plate and all reactions were run at room temperature under argon. NaBH_4_ (30.2 mg, 0.8 mmol) was dissolved in freshly deoxygenated water (100 mL). Citric acid (15.4 mg, 0.08 mmol) was added and the resulting solution was stirred for 60 min. During this period, the evolution of hydrogen gas was visible. Thereafter, CoCl_2_ in water (0.2 mL of a 0.04 M stock solution, 0.08 mmol of CoCl_2_) was added while stirring the solution vigorously, which turned dark during the reduction and the Co NP forming process.

The thus-prepared solution of Co NPs was poured in one batch to a solution of H_2_PtCl_6_ in deoxygenated water (30 mL) (15 mg H_2_PtCl_6_ × 6 H_2_O; 0.03 mmol) and stirred under argon for 30 min. Thereafter, the solution was exposed to air while stirring vigorously (approx. 1 h) to allow the oxidation of the remaining Co NPs. The hollow Pt nanospheres Pt_s_ were collected by centrifugation (3 times for 45 min at 250,000 rpm).

Synthesis of Pt_c_: The same reaction set-up as above was used, but now the Pt(VI) salt solution was added to the Co NPs via syringe pump.

NaBH4 (15.1 mg, 0.4 mmol) was dissolved in 100 mL deoxygenated water and a solution of citric acid (1 mL of 0.04 M solution in water, 0.04 mmol) was added. The resulting solution was stirred until H_2_ evolution was finished (10–20 min). Thereafter, a CoCl_2_ solution (1 mL of the 0.04 M stock solution, 0.04 mmol) was added via syringe over 10 min and stirred for 1 h at room temperature. The solution turned dark during the reduction and Co NP forming process. To the thus-prepared Co NP solution was added a solution of H_2_PtCl_6_ (0.75 mL of a 0.04 M stock solution of H_2_PtCl_6_ diluted to 30 mL with deoxygenated water) via syringe pump over a period of 2 h. After the completion of addition, the reaction mixture was stirred for another hour. Thereafter, it was exposed to air while stirring vigorously to oxidize all remaining Co NPs. The Pt hollow-sphere nanoparticles were collected by centrifugation (3 times for 45 min at 250,000 rpm). This material will be called Pt_c_ in the following sections.

For reasons of clarity, the materials from the two synthesis procedures will be denoted as Pt_s_ and Pt_c_, corresponding to the steep and continuous Pt salt addition to the Co-based solution during the synthesis, respectively.

### 4.2. TEM Characterization

The particles synthesized by the procedures described in Section 4.1 were characterized by TEM using a double-corrected JEOL^®^ ARM200-CFEG microscope (JEOL Ltd., Tokyo, Japan) operated at 200 kV. For TEM experiments under vacuum, particles were dispersed in ethanol and deposited on carbon membranes. High-angle annular dark-field scanning TEM (HAADF-STEM) images were recorded with a probe size of 0.9 nm and a probe current of 120 pA, using a convergence angle of 24~29 mrad for the micrographs acquisition. Acceptance angles for the inner diameter and outer diameter of 68 mrad and of 280 mrad, respectively, were used. Chemical analysis of the particles was carried out using energy-dispersive X-ray spectroscopy (EDS) using a JEOL^®^ JED detector with a collection angle of 1 sr.

The 3D morphology of the particles was investigated using electron tomography (ET). Prior to sample deposition, one drop of colloidal solution containing 5 nm Au nanoparticles solution was deposited on the TEM grid membrane followed by a plasma cleaning under an Ar–H_2_ gas mixture. Stacks of 1024 × 1024 px images were recorded in STEM-HAADF mode across an angular range of −68° to +68° with a step of 3° and with an exposure time of 10 µs per pixel. The acquisition of the tilt series was performed using single-axis geometry. The ImageJ software’s plugin, TomoJ V3 dual beta, was used for the series alignment using the Au nanoparticles as fiducial markers. The OS-SART reconstruction algorithm built in the TomoJ plugin was used for the volume reconstructions with 150 iterations and using a median filter throughout the volume. The Slicer3D version 4.11 software was employed for the data segmentation and modeling.

The thermal stability and catalytic behavior of the Pt-based catalysts were investigated by means of in situ approach experiments carried out in a closed-cell experimental device manufactured by Protochips: Atmosphere [11]. A pair of electron-transparent SiNx windows built up on SiC Echips were used to form the environmental cell (E-cell) with the sample placed on one of the windows, as a function of the working mode employed during the TEM investigation. Since the STEM was employed all along the current investigation, adapted sets of Echips were employed, such that the specimen was all the time placed in the upper side of the cell (the heating Echip). This configuration also allows the minimization of the beam and ionization effects on the specimen which are inevitable during the imaging, being at the same time adapted to the “analytical” STEM experimentations by EDS and/or electron energy-loss spectroscopy (EELS). In the Atmosphere set-up, the gas input and control are realized via a dedicated manifold connected to the sample holder, allowing to closely monitor the concentration and flow of gas mixtures all along the experiment. This gas manifold incorporates three reservoirs. Gas purging is achieved via a mechanical pump connected to the reservoirs and sample cell holder. Two experimental tanks deliver gases to the sample, while a third reservoir regulates pressure through a pressure differential mechanism. The vacuum in situ thermal treatment (in the TEM column vacuum) was carried out by using the same device as for the environmental experiments, in an effort to ensure a similar control of the temperature.

For the gas-assisted in situ TEM experiments, after a complete purge of the system with Ar at 100 Torr, a pressure of 1 bar of H2 was introduced into the experimental cell at a constant gas flow of 0.05 sccm (standard cm^3^/min). The initial state of the particles is observed under the argon atmosphere at 120 °C, a temperature at which the microstructure of the material to be investigated is not impacted. Heating the sample up to 120 °C under a neutral environment allows the desorption of ligands from the synthesis process from the sample surface, minimizing in this way their influence on the imaging process. During the temperature ramping, the electron beam was turned off in a trial to minimize the beam effect. The thermal treatment up to 600 °C, at a heating rate of 0.4 °C/s, was carried out as function of the material’s behavior upon gas exposure. The micrographs upon the in situ experiments were acquired under the very same conditions: probe size, current, exposure time, etc.

## 5. Conclusions

In conclusion, the thermal stability of Pt-based hollow nanospheres under vacuum and hydrogen flow at the atmospheric pressure was demonstrated by using an in situ environmental TEM approach. The galvanic replacement method employed for the materials’ synthesis led to the development of HNSs with outer mean diameters of 16 nm and 34 nm, corresponding to the addition of Pt salts in one step or at a continuous constant rate, respectively. The as-synthesized nanostructures contain small amounts of Co from the templating particles used in the synthesis. The HNS outer surface is marked by the presence of nanoparticles (2–3 nm) constituting the HNS shells, whereas their inner surface exhibits well-defined facets. Due to the presence of open channels within the HNS shells, these types of HNSs can be foreseen as nanoreactors for well-defined catalytic reactions. The in situ TEM treatment under vacuum and hydrogen flow demonstrated the high thermal stability of the HNSs, up to more than 500 °C. The microstructural evolution of HNSs under thermal constraints involves two steps: the development of a continuous shell by the diffusion and sintering of the nanoparticles from the HNS crown and the nanosphere collapse, marked by the transition from the hollow to solid morphology. The continuous shell formation is complete at lower temperatures in the case of smaller HNSs no matter the reaction environment. However, in the presence of hydrogen, the temperatures needed for the shell to attain a quasi-constant thickness are lower than under vacuum. The process of HNSs collapsing begins with the diminishing of the inner cavity size and continues until the morphology becomes completely solid. As such, the early collapse of the Pt-based hollow nanospheres under vacuum as compared to the hydrogen environment was evidenced and associated to both the presence of channels within the shell and the hydrogen-reactive environment. This accumulation of factors induces a strong faceting of the nanospheres’ inner surface, which renders the hollow nanospheres highly stable. The main achievement of this study was to demonstrate the reliability of the in situ TEM methodology to explore the microstructural stability of Pt-based hollow nanospheres under two fundamentally different environments: the neutral TEM vacuum and the highly reactive hydrogen environment at high pressure (1 bar) and well-controlled flow. The Pt hollow nanospheres synthesized in this work can be successfully assimilated to nanoreactors that can find applications in multiple catalytic reactions, particularly under reductive environments.

## Figures and Tables

**Table 1:** Synthesis of the Pt_c_ and Pt_s_ HNS characteristics as revealed by the TEM measurements.

HNS Type	HNS Diameter [nm]/Arrangement	NPs Diameter [nm]	Shell Thickness [nm]	Channel Number/HNS	Channel Diameter [nm]	Co Content [at. %]
Pt_S_	16 ± 4/agglomerates	3 ± 1	4 ± 1	5 ± 2	3 ± 2	13 ± 5
Pt_C_	34 ± 4/chains	2 ± 1	5 ± 2	14 ± 2	8 ± 2	7 ± 3

## Data Availability

Data presented in this study are available on request from the authors.

## Supplementary Materials

The following supporting information can be downloaded at https://www.mdpi.com/article/10.3390/molecules30040792/s1. Figure S1. EDS-STEM line analysis of the local composition within an individual HNS from Pts specimen showing the intensities of both the Pt and Co, as well as their ratio. Figure S2. (a) STEM-HAADF micrograph showing the HNS chains assemblies within the PtC, (b) Histogram displaying the chains length distribution. pecimen showing the intensities of both the Pt and Co, as well as their ratio. Figure S3. Thermal evolution of PtS HNS under vacuum. (a) STEM-HAADF micrographs of a typical region and (b) individual HNS with the intensity profiles along the arrows; the yellow arrows: HNS prior and after the NPs coalescence, the green arrows : the open channels and the red circles: the surface topology. Figure S4. Thermal evolution of Pts HNS under TEM vacuum. (a) micrographs of the very same region acquired for temperatures from 120 °C to 550 °C; scalebar: 20 nm. Figure S5. Thermal evolution of Ptc HNS under TEM vacuum; micrographs of the very same region acquired for temperatures from 120 °C to 550 °C; scalebar: 50 nm. Figure S6: Thermal evolution of Pts hollow nanospheres under hydrogen flow. (a,b) STEM-HAADF micrographs of typical region and of a single HNS with the corresponding intensity ptofile along the arrows; (c) Pt and Co signals redrawn from the EDS line scan acquired after the H2 treatment at 300 °C. Figure S7. Thermal evolution of Pts HNS under hydrogen flow; micrographs of the very same region acquired for temperatures from 120 °C to 400 °C; scalebar: 20 nm. Figure S8. Thermal evolution of Ptc HNS under hydrogen flow. (a) micrographs of the very same region acquired for temperatures from 120 °C to 550 °C; scalebar: 50 nm. Figure S9. (a) The methodology used for the measurement of the interparticular distance x, as schematically defined in (b). (c) Evolution of the measured distances x with the temperature, with the mean activation energies estimated from the data by considering an Arrhenius-type law. Figure S10. Histograms of particles size distribution determined corresponding to the vacuum and hydrogen assisted thermal treatment of the Pts HNS at an intermediary temperature, 300 °C and after HNS collapse at 550 °C.
