# Supplementary material for: Insights on Morphology and Thermal Stability of Hollow Pt Nanospheres by In Situ Environmental TEM
Source: Molecules. 2025 Feb 8;30(4):792. doi: 10.3390/molecules30040792 (PMC11858631; doi:10.3390/molecules30040792)
Supplement: Supplementary file 1 [file molecules-30-00792-s001.zip › molecules-3429116-supplementary.pdf]

Supplementary Information

Insights on morphology and thermal stability of hollow Pt nano-spheres by in-situ Environmental TEM

Josephine Rezkallah[1, 3], Xavier Sauvage[1], Bernhard Witulski[2], Simona Moldovan[1, *]

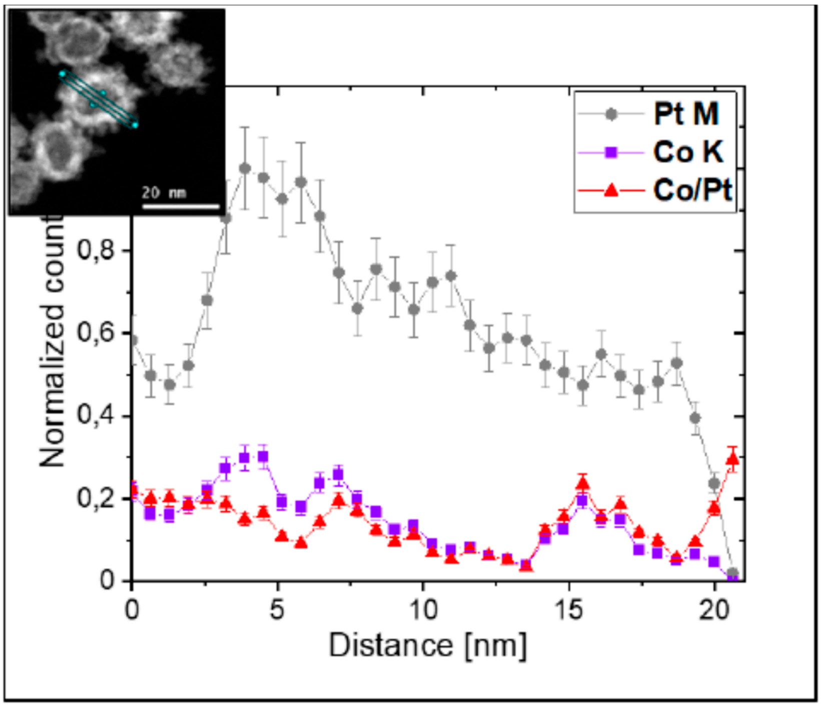

Figure S1. EDS-STEM line analysis of the local composition within an individual HNS from Pt_S specimen showing the intensities of both the Pt and Co, as well as their ratio

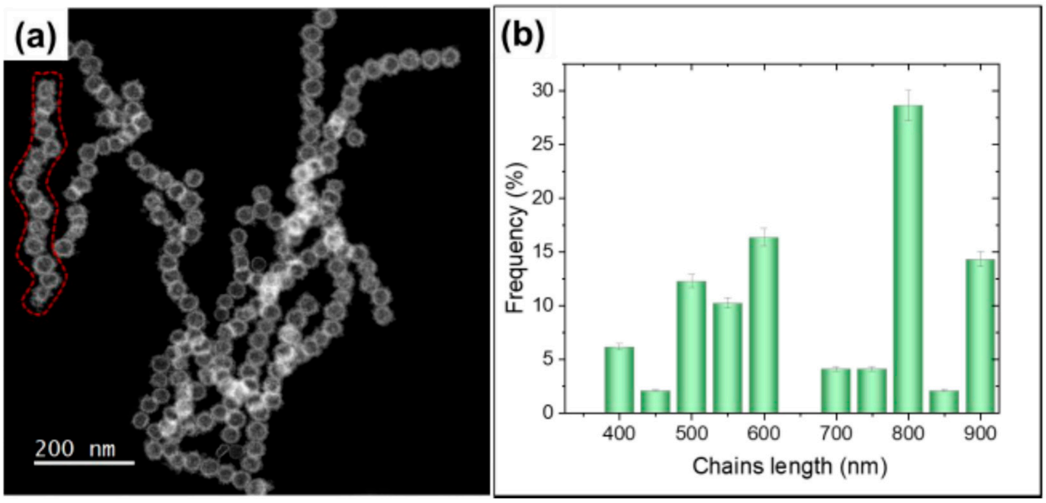

Figure S2. (a) STEM-HAADF micrograph showing the HNS chains assemblies within the Pt_C, (b) Histogram displaying the chains length distribution.

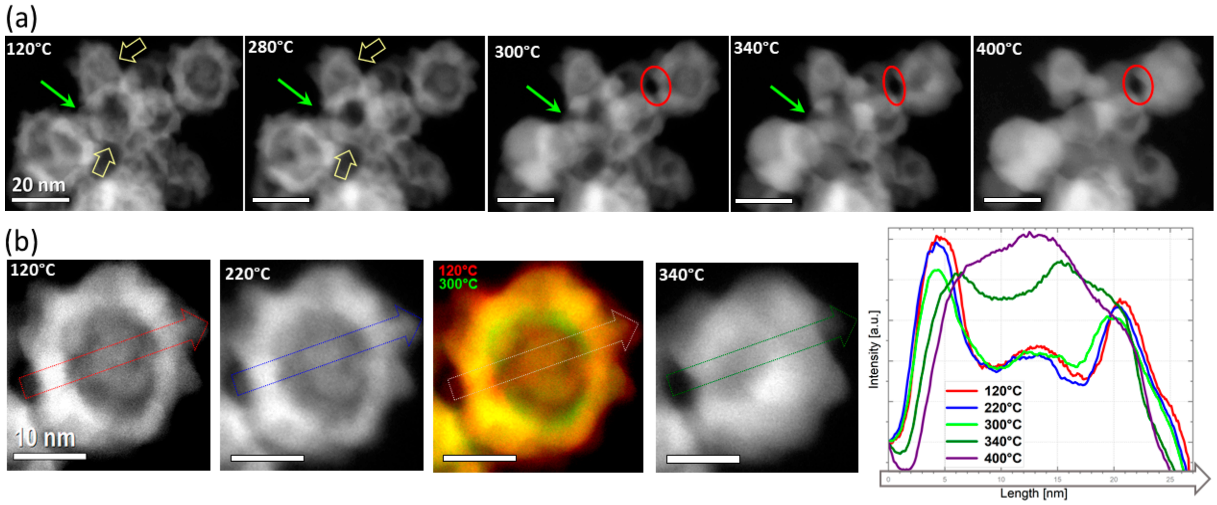

Figure S3. Thermal evolution of Pt_s HNS under vacuum. (a) STEM-HAADF micrographs of a typical region and (b) individual HNS with the intensity profiles along the arrows; the yellow arrows: HNS prior and after the NPs coalescence, the green arrows : the open channels and the red circles: the surface topology

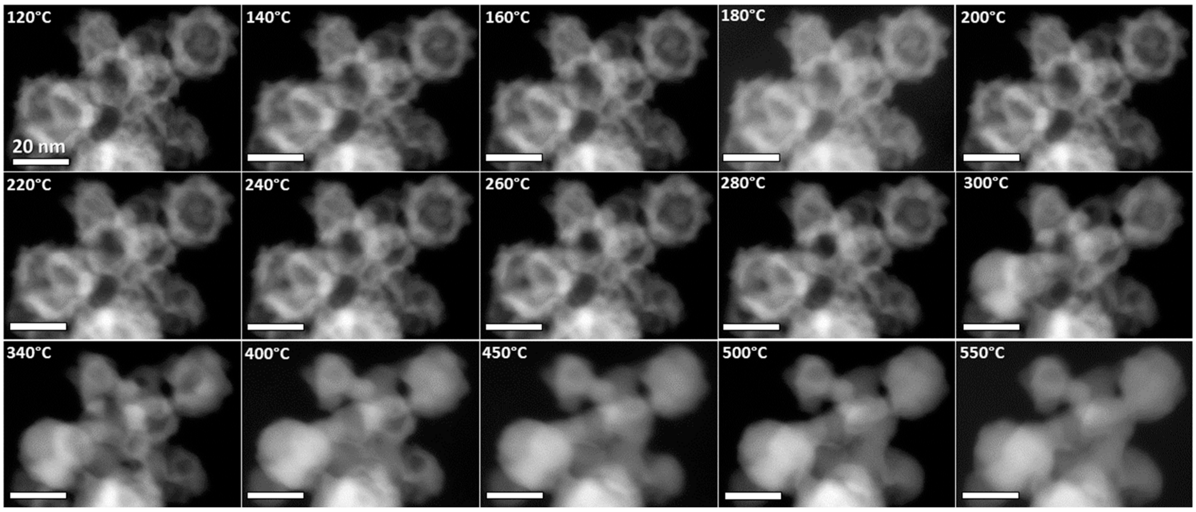

Figure S4. Thermal evolution of Pt_s HNS under TEM vacuum. (a) micrographs of the very same region acquired for temperatures from 120°C to 550°C; scalebar: 20 nm

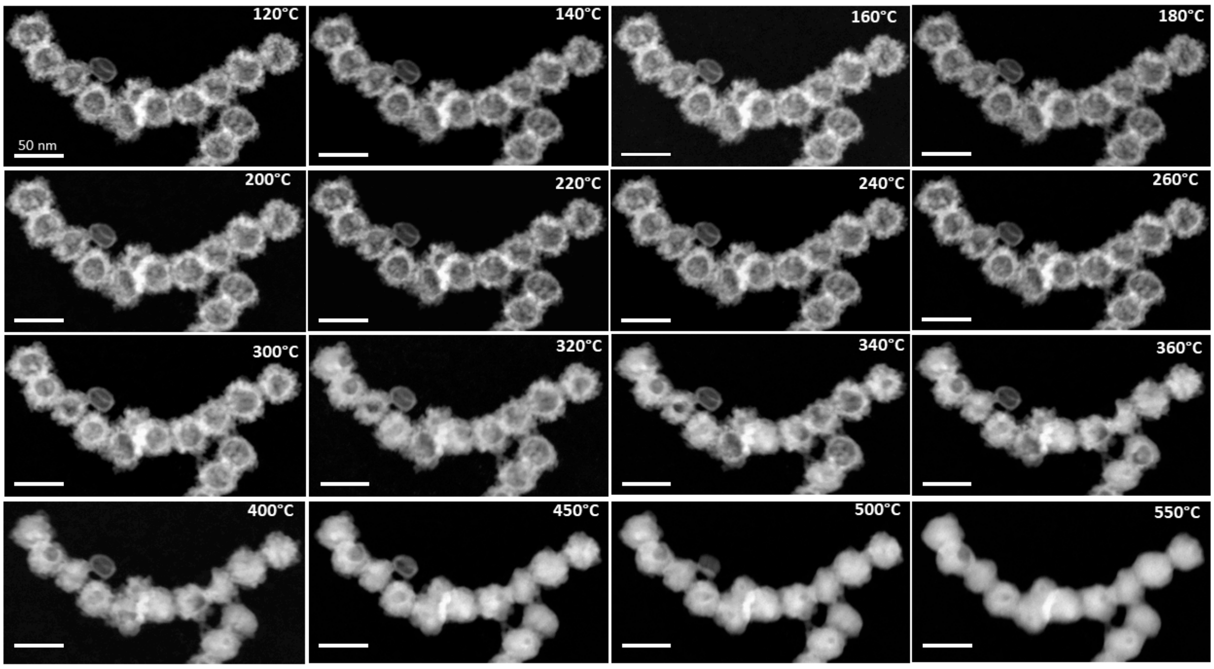

Figure S5. Thermal evolution of Pt_c HNS under TEM vacuum; micrographs of the very same region acquired for temperatures from 120°C to 550°C; scalebar: 50 nm

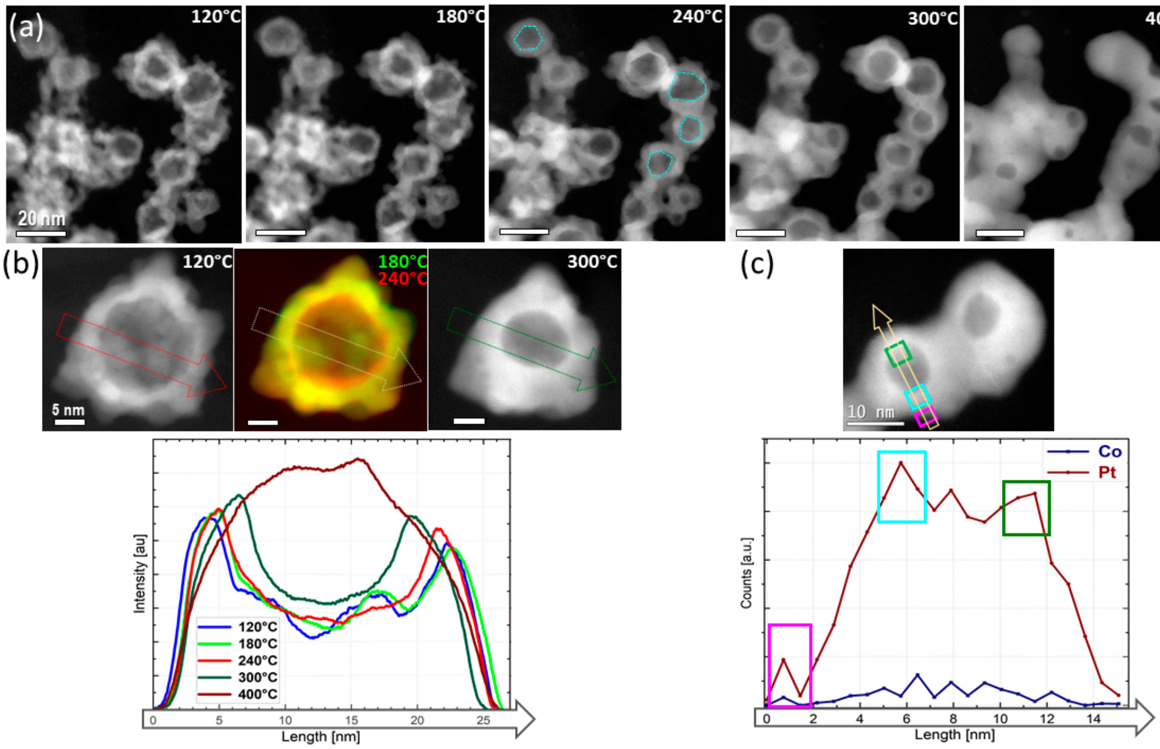

Figure S6: Thermal evolution of Pt_s hollow nanospheres under hydrogen flow. (a)- (b) STEM-HAADF micrographs of typical region and of a single HNS with the corresponding intensity ptofile along the arrows; (c) Pt and Co signals redrawn from the EDS line scan acquired after the H_2 treatment at 300°C.

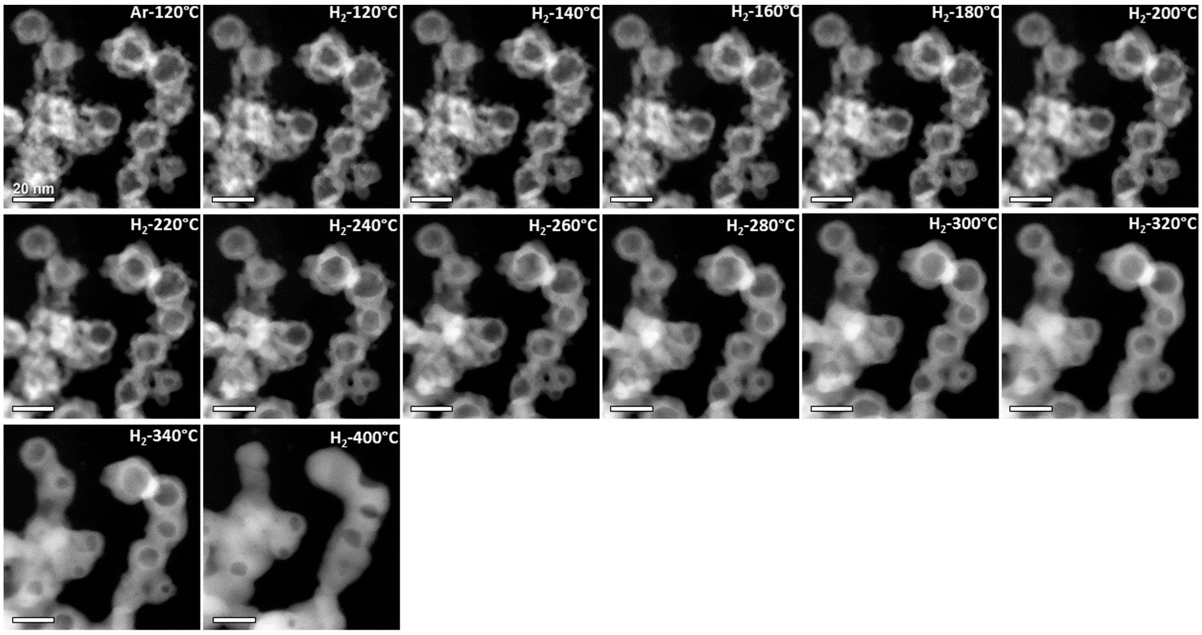

Figure S7. Thermal evolution of Pt$_s$ HNS under hydrogen flow; micrographs of the very same region acquired for temperatures from 120°C to 400°C; scalebar: 20 nm

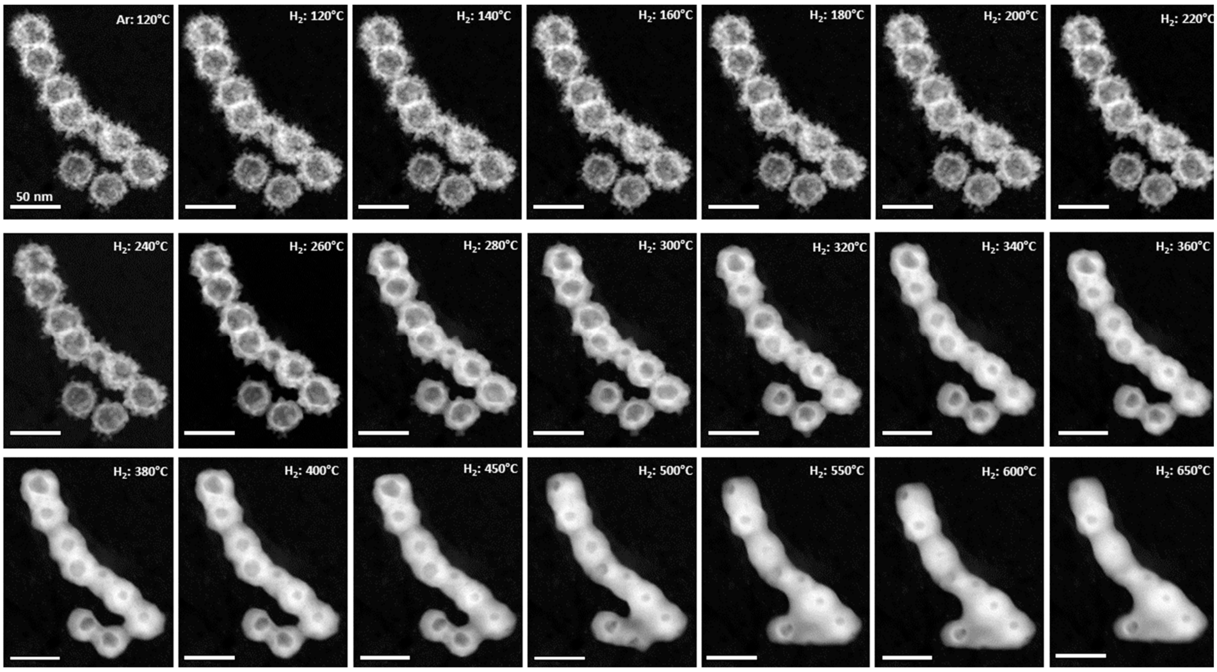

Figure S8. Thermal evolution of Pt$_c$ HNS under hydrogen flow. (a) micrographs of the very same region acquired for temperatures from 120°C to 550°C; scalebar: 50 nm

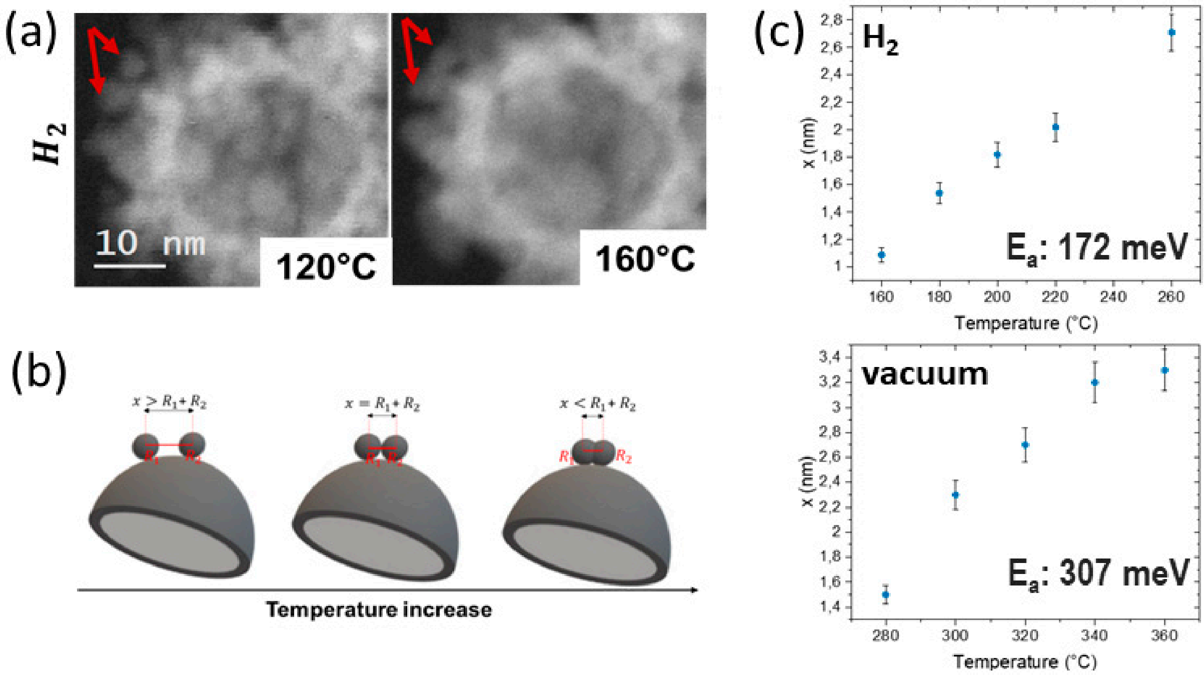

Figure S9. (a) The methodology used for the measurement of the interparticular distance x, as schematically defined in (b). (c) Evolution of the measured distances x with the temperature, with the mean activation energies estimated from the data by considering an Arrhenius-type law

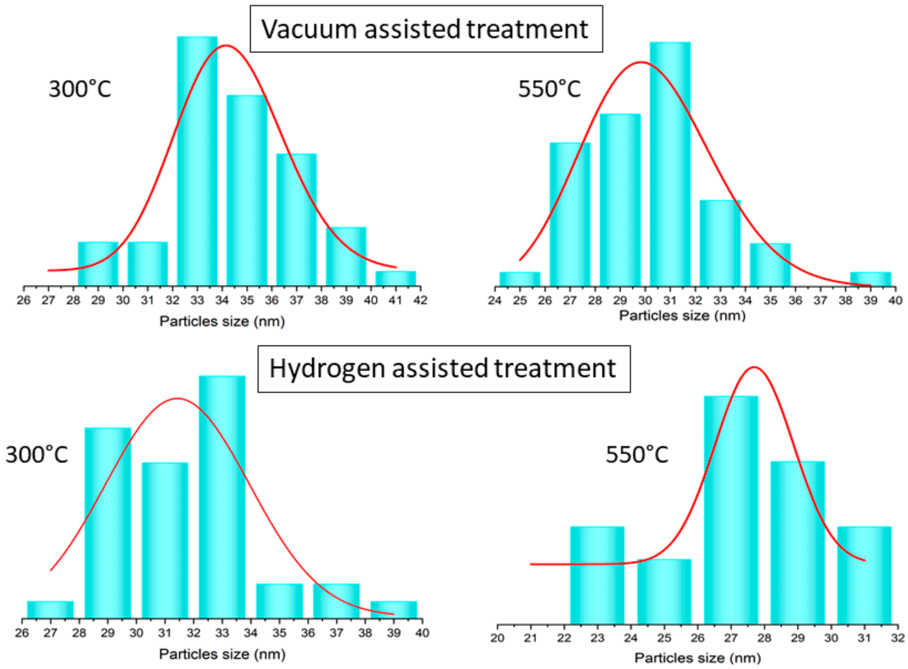

Figure S10. Histograms of particles size distribution determined corresponding to the vacuum and hydrogen assisted thermal treatment of the Pt_s HNS at an intermediary temperature, 300°C and after HNS collapse at 550°C
